# Sensitive assay design for detection of anti-drug antibodies to biotherapeutics that lack an immunoglobulin Fc domain

**DOI:** 10.1038/s41598-021-95055-x

**Published:** 2021-07-29

**Authors:** Derrick Johnson, Erica Simmons, Sanofar Abdeen, Adam Kinne, Elijah Parmer, Sherri Rinker, Jennifer Thystrup, Swarna Ramaswamy, Ronald R. Bowsher

**Affiliations:** B2S Life Sciences LLC, 97 East Monroe Street, Franklin, IN 46131 USA

**Keywords:** Biotechnology, Biological techniques, Immunological techniques, ELISA

## Abstract

Today the evaluation of unwanted immunogenicity is a key component in the clinical safety evaluation of new biotherapeutic drugs and macromolecular delivery strategies. However, the evolving structural complexity in contemporary biotherapeutics creates a need for on-going innovation in assay designs for reliable detection of anti-drug antibodies, especially for biotherapeutics that may not be well-suited for testing by a bridging assay. We, therefore, initiated systematic optimization of the direct binding assay to adapt it for routine use in regulatory-compliant assays of serum anti-drug antibodies. Accordingly, we first prepared a SULFO-TAG labeled conjugate of recombinant Protein-A/G to create a sensitive electrochemiluminescent secondary detection reagent with broad reactivity to antibodies across many species. Secondly, we evaluated candidate blocker-diluents to identify ones producing the highest signal-to-noise response ratios. Lastly, we introduced use of the ratio of signal responses in biotherapeutic-coated and uncoated wells as a data transformation strategy to identify biological outliers. This alternative data normalization approach improved normality, reduced skewness, and facilitated application of a parametric screening cut point. We believe the optimized direct binding assay design employing SULFO-TAG labeled Protein-A/G represents a useful analytical design for detecting serum ADA to biotherapeutics that lack an immunoglobulin Fc domain.

## Introduction

Today, the evaluation of unwanted immunogenicity is an integral component in the overall clinical safety assessment of new candidate biotherapeutic drugs and macromolecular delivery strategies^1–4^. In addition much progress has been made over the past two decades in devising a harmonized testing strategy for detection and characterization of anti-drug antibodies (ADA) to support investigations of unwanted immunogenicity^5–7^. This collaborative effort has culminated in a multi-tiered testing paradigm that is described widely in publications^5,6,8,9^ and guidance documents from global regulatory agencies^10–12^. Yet, despite having a consensus approach and availability of multiple assay design options^13–15^, anti-drug antibody testing remains a challenging endeavor owing to the increasing diversity and structural complexity in modern biotherapeutics.

Over the last decade monoclonal antibodies (mAbs) have comprised a high percentage of the biotherapeutics undergoing development and achieved notable clinical successes across multiple disease categories, including autoimmune disorders^16,17^ and cancer^18–20^. Because of the widespread interest in therapeutic mAbs much attention has focused on refining the analytical methodology for detection and characterization of unwanted ADA to this class of drugs^5,8,9,21^. Accordingly, for multiple reasons, including appreciable homology with unrelated serum immunoglobulins, ADA testing of mAbs relies heavily on the ‘bridging’ assay design^5,8,9,22^. This assay design takes advantage of paratope-specific interactions between a bivalent serum ADA and labeled versions of the monoclonal antibody therapeutic (i.e., often a biotinylated version for solid-phase capture reagent and a different labeled form for detection) to form a bridging complex in which the resultant signal response is proportional to the concentration of ADA in the serum test sample. Notwithstanding the commonly encountered technical challenges of drug tolerance^22,23^ and/or target interference^24^, bridging assays possess numerous attributes that make them appealing for use in ADA detection. These include low nonspecific background, good assay sensitivity, indifference to detection of surrogate antibodies from different mammalian species, efficient detection of immunoglobulin isotypes and operational ease for high throughput^13–15^. Hence, the bridging assay is at present the predominant analytical design used for detecting unwanted ADA during clinical evaluation of many candidate biotherapeutic drugs.

Despite widespread use of the bridging assay, not all contemporary biotherapeutics are well-suited for ADA detection by this assay design. This fact has spurred on-going research into the development of alternative assay designs for reliable ADA detection to meet analytical performance characteristics outlined in regulatory guidance documents. Some notable examples include the affinity-capture-and-elution assay^25,26^ (i.e., ACE methodology) and the precipitation-and-acid-dissociation assay^27^ (i.e., PandA methodology). Since both assay designs are highly drug tolerant, they have gained popularity for detecting ADA to mAb therapeutics that have a long terminal elimination half-life.

The direct binding assay represents another assay approach for detecting unwanted ADA. However, despite being conceptually straightforward and easy to execute for high sample throughput, the direct binding assay has several characteristics that heretofore have rendered it less desirable as an assay design for supporting clinical ADA testing^13–15^. These include a tendency for having high serum background responses that result in unsatisfactory sensitivity, being prone to increased inter-subject variability that can result in high assay cut points, and most notably being cumbersome to perform because of the need for using multiple species-specific detection reagents. Consequently, these issues have tended to discourage use of this assay design in clinical drug development and have relegated it to being a back-up strategy when other ADA assay designs prove unsuccessful. Accordingly, we undertook optimization of the direct binding assay design to overcome its perceived limitations and to adapt it for routine use in detecting unwanted ADA.

In this report we systematically addressed the issues of high matrix background, inadequate sensitivity, high inter-subject (biological) variability and the requirement to detect multiple classes of immunoglobulins from both human and surrogate animals. We offer a versatile assay design strategy that is sensitive, useful for supporting clinical testing, and amenable for application with a wide range of contemporary biotherapeutics that lack an immunoglobulin Fc domain. In brief, we developed and validated a direct binding assay design in which the biotherapeutic-of-interest is first coated on a MSD plate and functions to capture reactive antibodies in diluted serum test samples. After capture SULFO-TAG labeled Protein-A/G is used a single multi-species reagent for detecting bound immunoglobulins. The labeled reagent offers excellent sensitivity for immunoglobulin detection by Meso Scale Discovery (MSD) electrochemiluminescence technology^22,25^, while MSD plate blocking and sample dilution with an optimal reagent, such as ChonBlock^26,27^, Assay Diluent or Blocker casein, reduces serum background signal responses that enhance specific binding (signal-to-noise). In addition, we implemented the use of biotherapeutic-coated and uncoated MSD plate wells as a novel strategy for improved normalization of test sample signal responses. In assays employing detection with SULFO-TAG labeled Protein-A/G this data transformation reduces inter-subject variability and increases distributional normality which facilitates application of parametric ADA screening cut points. In combination, these innovations culminated in a versatile direct binding assay design that is sensitive, easy to execute and well-suited to support regulatory-compliant detection of ADA in clinical evaluations of unwanted immunogenicity for biotherapeutics that lack an immunoglobulin Fc domain.

## Results

Protein-A/G was conjugated with SULFO-TAG as described in the “Materials and methods” section. After purification, the labeled product underwent detailed characterization that included purity assessment by HPLC-SEC, measurement of concentration (BCA Protein Assay), and estimation of the SULFO-TAG label molar incorporation ratio. Based on its chromatographic profile, the protein conjugate eluted at a retention time of 8.99 min (flow rate of 1 mL/min) with purity > 99%. The incorporation ratio in the final product was 7 mol of SULFO-TAG per mol of Protein-A/G.

**Figure 1:**
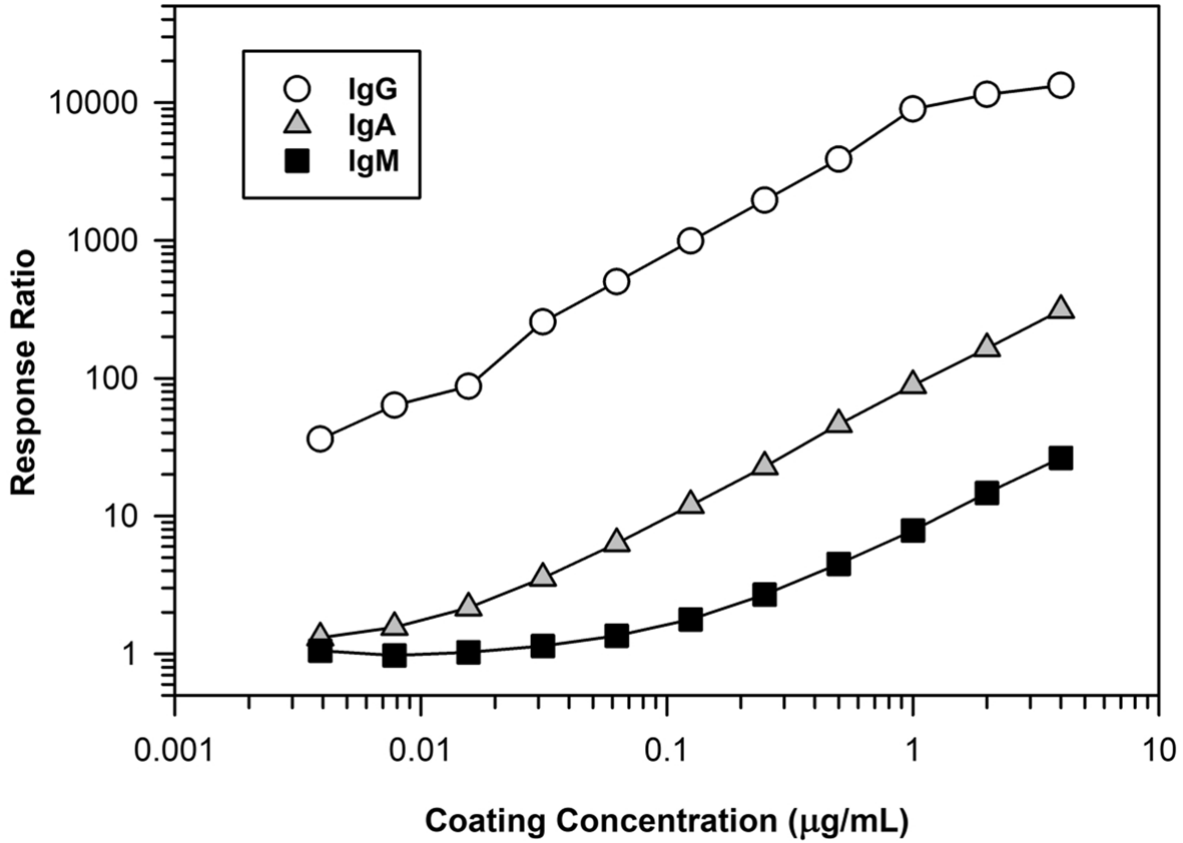
Detection of different human immunoglobulin classes by SULFO-TAG Protein-A/G. A MSD plate was coated with varying concentrations of human IgG, IgM, or IgA diluted in PBS and incubated it overnight at 4 °C. On the following day, the plate was washed and blocked with PBS-2% BSA. After another wash, the coated immunoglobulins were detected using 0.2 µg/mL SULFO-TAG labeled Protein-A/G diluted in PBS-2% BSA. The plot shows the ratio of the relative luminescence (RLU) signal responses for coated wells relative to uncoated wells.

We evaluated the purified SULFO-TAG labeled Protein-A/G to verify its utility as a versatile reagent to detect different classes of human immunoglobulins. As shown in Fig. 1, we confirmed this reagent did detect different classes of human immunoglobulins, albeit with greater reactivity towards IgG. Despite the higher reactivity with IgG, both IgM and IgA displayed adequate signal responses to permit use of this labeled reagent for detecting these ADA isotypes.

**Figure 2:**
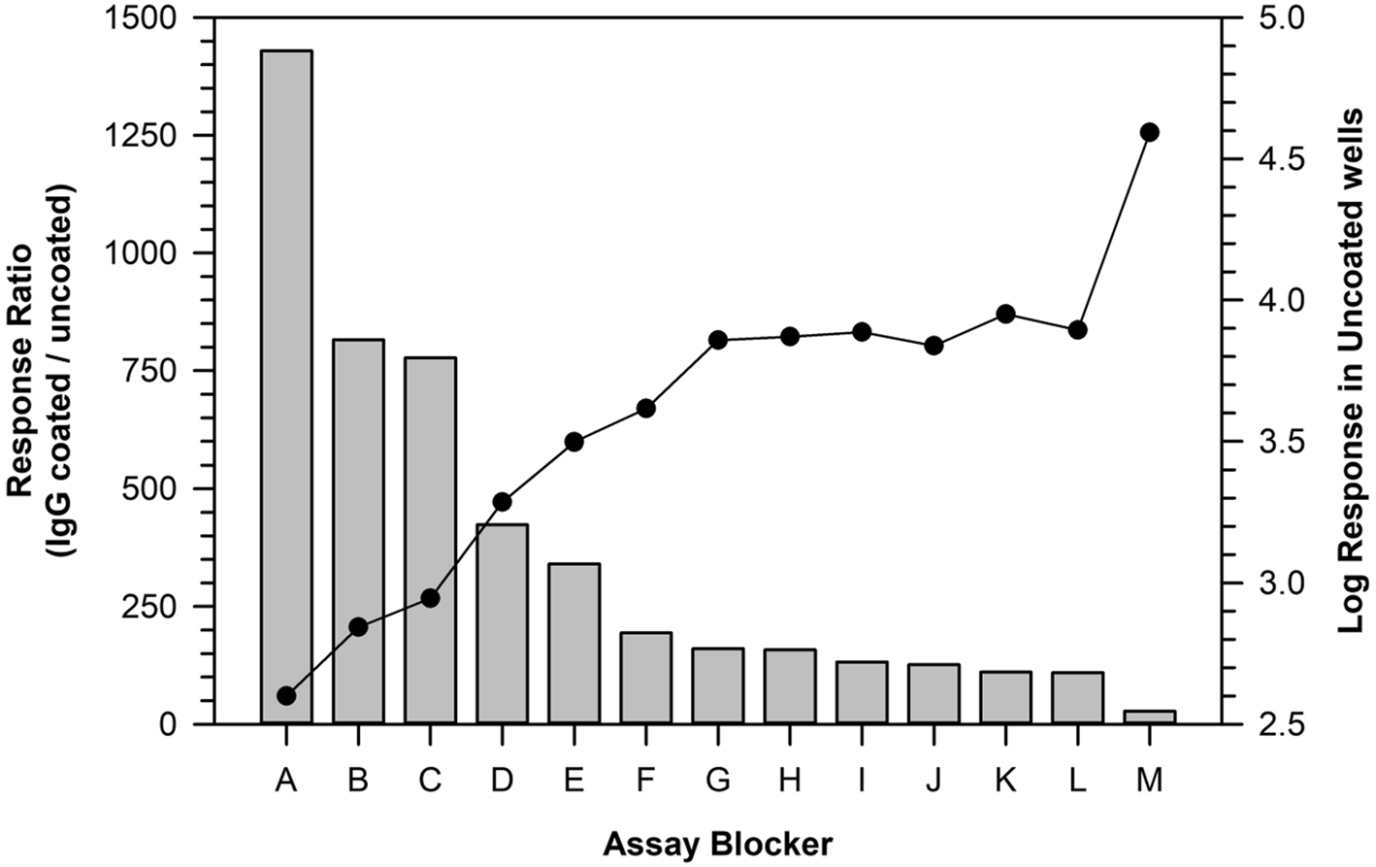
Effectiveness of different plate blocker and sample diluents for use in detection of serum immunoglobulins by SULFO-TAG labeled Protein-A/G. MSD plate wells were coated overnight with either 2 µg/mL human IgG or PBS (uncoated). On the following day, the wells were block using the different blocker-diluent buffers (A through M). A lot of pooled human serum was diluted 1/50 in the various blocker-diluents, added to the plate wells in duplicate and incubated at ambient temperature. After 1 h the plate was washed, and the IgG was detected by incubation with 0.2 µg/mL of SULFO-TAG labeled Protein-A/G diluted in PBS-2% BSA. The response ratio (bar graph) is RLU responses from the IgG coated wells relative to uncoated wells. Assay Blocker A is ChonBlock, B is Assay Diluent, C is Blocker casein in PBS, D is Low Cross buffer, and E is Monster Block. Blockers F through L, which produced similar response ratios, consisted of Starting Block, Super Block, Neptune Block, SynBlock and various combinations of BSA/HSA with PBS and TBS. The least effective blocker, M, was PBS alone. Closed circles depict the Log RLU from the uncoated wells and shows the wide range in non-specific binding responses seen among the different diluent blockers.

Upon demonstration of suitable cross-reactivity towards different human immunoglobulin isotypes, we used the SULFO-TAG labeled Protein-A/G reagent in an experiment to screen a series of blocker-diluents for their potential utility to optimize specific detection of reactive serum antibodies (i.e., the ratio in signal responses between biotherapeutic-coated and uncoated MSD wells). As shown in Fig. 2, ChonBlock (Chondrex) was the most efficient reagent for maximizing the response ratio, while Assay Diluent (Suromodics) and Blocker casein (ThermoFisher) were also quite effective. The least efficient blocker-diluents were PBS/TBS buffers supplemented with BSA or HSA which produced response ratios about 15-times lower than ChonBlock. For all candidate blocker-diluents we noted that their effectiveness was dictated largely by their ability to limit the non-specific binding signal responses in uncoated wells.

**Figure 3:**
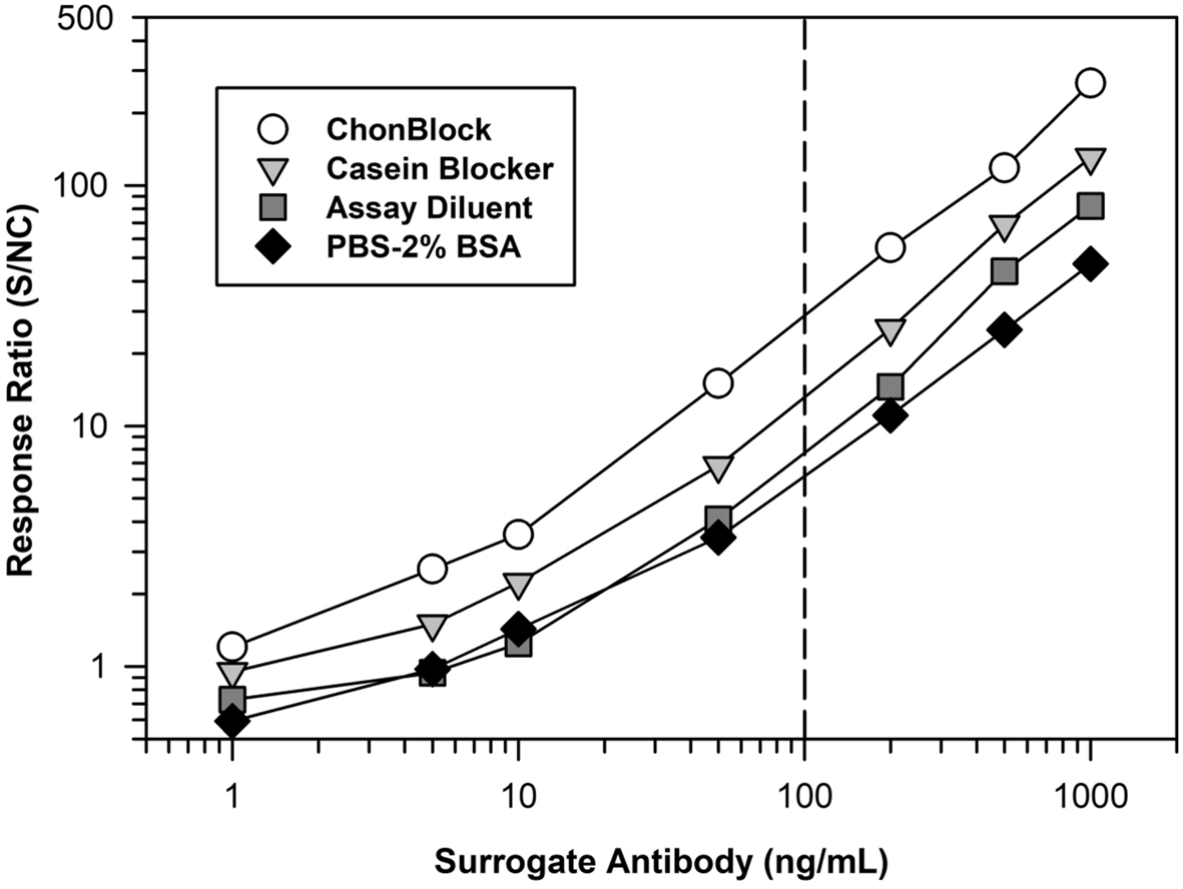
Sensitivity comparison of the optimal blocker-diluents for detection of serum ADA. On day 1 the MSD plate was coated overnight at 4 °C with 2 µg/mL of a representative biotherapeutic. On day 2, the plate was washed and blocked with the various candidate blocker-diluents. A standard curve of a surrogate specific rabbit antibody was prepared in human serum and then diluted 1/100 in the various buffers. The assay was performed, as described in the “Materials and methods” section, using detection with 0.1 µg/mL of SULFO-TAG labeled Protein-A/G diluted in LowCross Buffer. The reported response ratio is the RLU of the surrogate antibody serum calibrators/RLU from the NC (i.e., human serum pool not supplemented with surrogate antibody).

Upon establishing the rank order for the effectiveness of candidate blocker-diluents, the optimal ones were evaluated further for their ability to detect serum ADA at the clinically meaningful concentration of 100 ng/mL^10^. Accordingly, Fig. 3 shows the concentration–response relationship for a serum standard curve of surrogate antibody diluted in the various blockers. Using a sample dilution of 1/100, both ChonBlock and Blocker casein generated response ratios > 10-times higher in samples supplemented with surrogate antibody at 100 ng/mL relative to unsupplemented matrix. These results verified the benefit of using SULFO-TAG labeled Protein-A/G with dilution in Chonblock (or Blocker casein) as an effective strategy for ADA detection.

Upon additional experimentation to evalute the minimal required dilution (MRD), a balance between maximizing analyte detection and minimizing matrix interference^7^, optimal antibody detection at 100 ng/mL was achieved at a serum MRD from 1/50 to 1/100 using ChonBlock. Similar results were obtained after dilution in either 100% ChonBlock (undiluted) or 50% ChonBlock (diluted 1:2 in buffer). We determined this workflow consistently offers reliable detection of serum ADA to biotherapeutics that lack an immunoglobulin Fc domain, the structural feature that binds to Protein-A/G^28,29^.

During method development we noted a high incidence of inter-subject outliers (i.e., samples showing a high screening signal response, but modest competitive % inhibition after adding the biotherapeutic-of-interest). Accordingly, we hypothesized the outliers were samples that had a high level of non-specific binding due to inter-subject differences in immunoglobulin concentrations or immune complexes which can adhere avidly to plastic surfaces and subsequently react with labeled Protein-A/G. To help mitigate this issue, we implemented use of the ratio of signal responses from biotherapeutic-coated and uncoated MSD wells, as an alternative to the commonly used data transformation approach of the ratio of sample signal response to a serum NC pool for calculating a screening cut point^30,31^. As reported in Table 1, data transformation with the Log of the ratio of signal responses from biotherapeutic-coated to uncoated wells yielded superior results in comparison to the conventional approach with improved normality (Shapiro–Wilk p-value) and reduced outliers that yield decreased right tail skewness. For each biotherapeutic the alternate normalization approach resulted in a normal distribution after only 1 round of outlier removal which permitted use of a model-based parametric cut point. In contrast, the standard normalization approach of Log (S/NC) yielded normality for only one of the three candidate biotherapeutics with all displaying higher skewness and data distributions having reduced central tendency that are better suited for application of a nonparametric screening cut point.

**Table 1:** Impact of different data transformations on distributional properties for resultant ADA tier 1 screening cut points.

Biotherapeutic	All data (no outliers removed)			Data (after outlier removal)^a^		
Data transformation^b^	(n) mean	Shapiro–Wilk test (p-value)	Skewness	Mean (n)	Shapiro–Wilk test (p-value)	Skewness
**Biotech 1**	(42)					
Log (S/NC)	0.207	< 0.0001	2.608	0.141 (38)	0.0138	0.820
Log (coated/uncoated)	− 0.133	< 0.0001	− 1.454	0.131 (35)	0.2150*	0.543
**Biotech 2**	(42)					
Log (S/NC)	0.273	0.2086*	0.507	^c^N.A	N.A	N.A
Log (coated/uncoated)	0.276	0.4637*	0.446	N.A	N.A	N.A
**Biotech 3**	(48)					
Log (S/NC)	0.354	< 0.0001	1.686	0.275 (46)	0.0091	0.824
Log (coated/uncoated)	0.423	< 0.0001	1.972	0.310 (45)	0.3979*	0.478

^a^Outliers were removed using outlier box plots in JMP (SAS institute, ver. 15.2.1).

^b^Individual results obtained by the direct binding assay described in the “Materials and methods” section underwent data transformation using Log (S/NC) and Log (coated wells/uncoated wells).

^c^N.A., not applicable. Initial data sets were normal and, thus, did not require outlier removal.

*Indicates the sample distribution is normal by Shapiro–Wilk test (p > 0.05).

**Figure 4:**
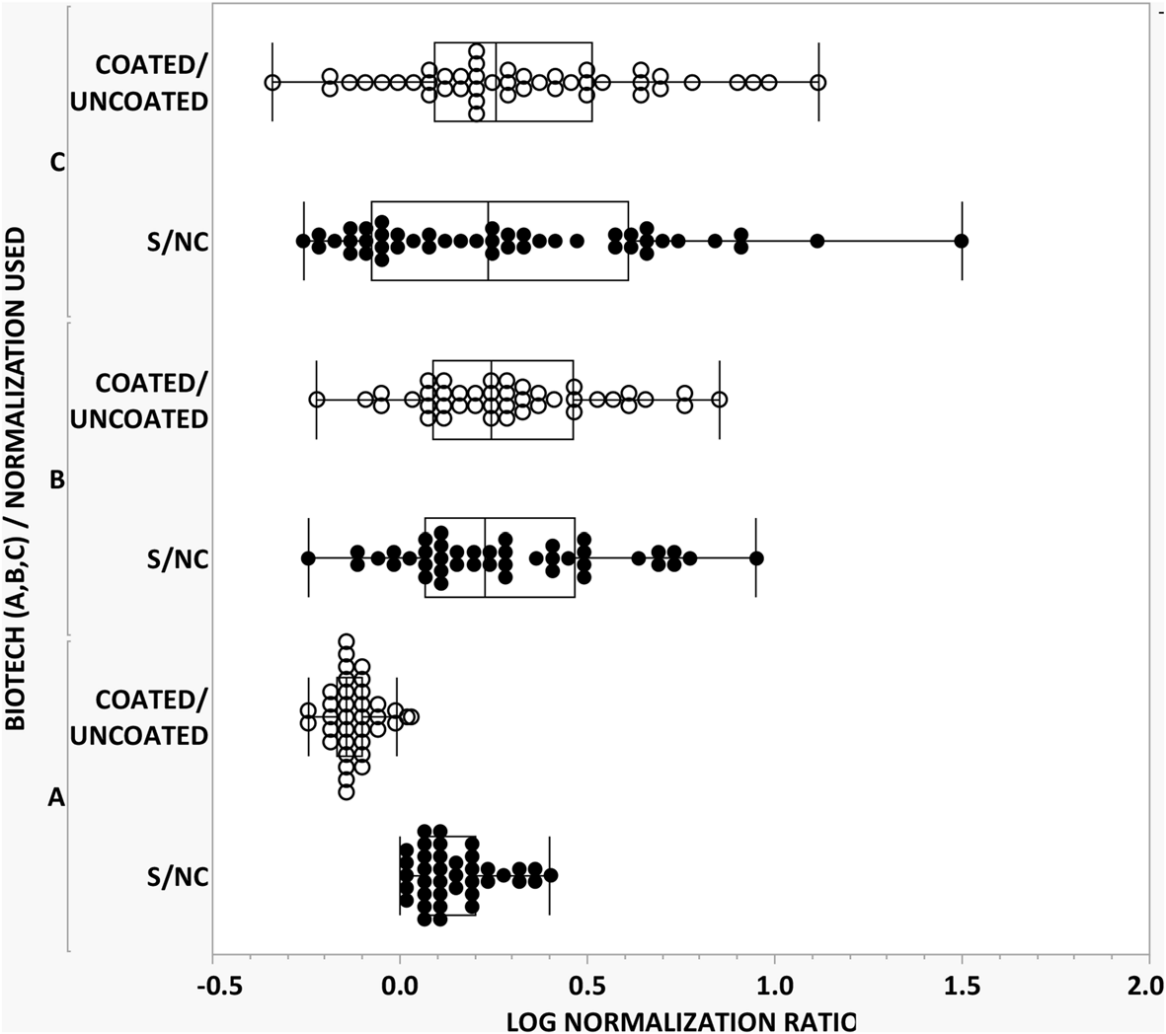
Comparison of different data transformation strategies for normalization of ADA signal responses. Three candidate biotherapeutic drugs were evaluated for determination of an ADA screening cut point. Individual signal responses were normalized by both the conventional approach involving S/NC (closed circle) and the modified approach using coated/uncoated wells (open circle) and then subjected to one round of outlier removal using box plot analysis (JMP, SAS institute, ver. 15.2.1). Results are reported in Table 1. As shown above the data transformation involving coated/uncoated wells yielded superior distributional normality with decreased skewness.

The positive impact of the alternative normalization approach compared to the Log (S/NC) on the various data distributions is also shown in Fig. 4. For at least two of the three biotherapeutics, application of the Log (S/NC) data transformation yielded normalized response distributions that showed poor central tendency combined with evidence for a long right tail. This scenario is better suited for application of a nonparametric screening cut point. In contrast, use of the ratio of signal responses from coated and uncoated wells for data transformation resulted in much improved central tendency. For all three biotherapeutics a higher number of results occurred within each box plot’s interquartile range combined with decreased right tail skewness. This pattern of normalized signal responses is well-suited for establishment of a desired model-based parametric screening cut point. Consequently, we believe the modified data transformation approach employing biotherapeutic-coated and uncoated wells warrants further consideration as a useful strategy for assay plate normalization in direct binding assay designs that employ a labeled secondary detection reagent, such as SULFO-TAG Protein-A/G.

**Figure 5:**
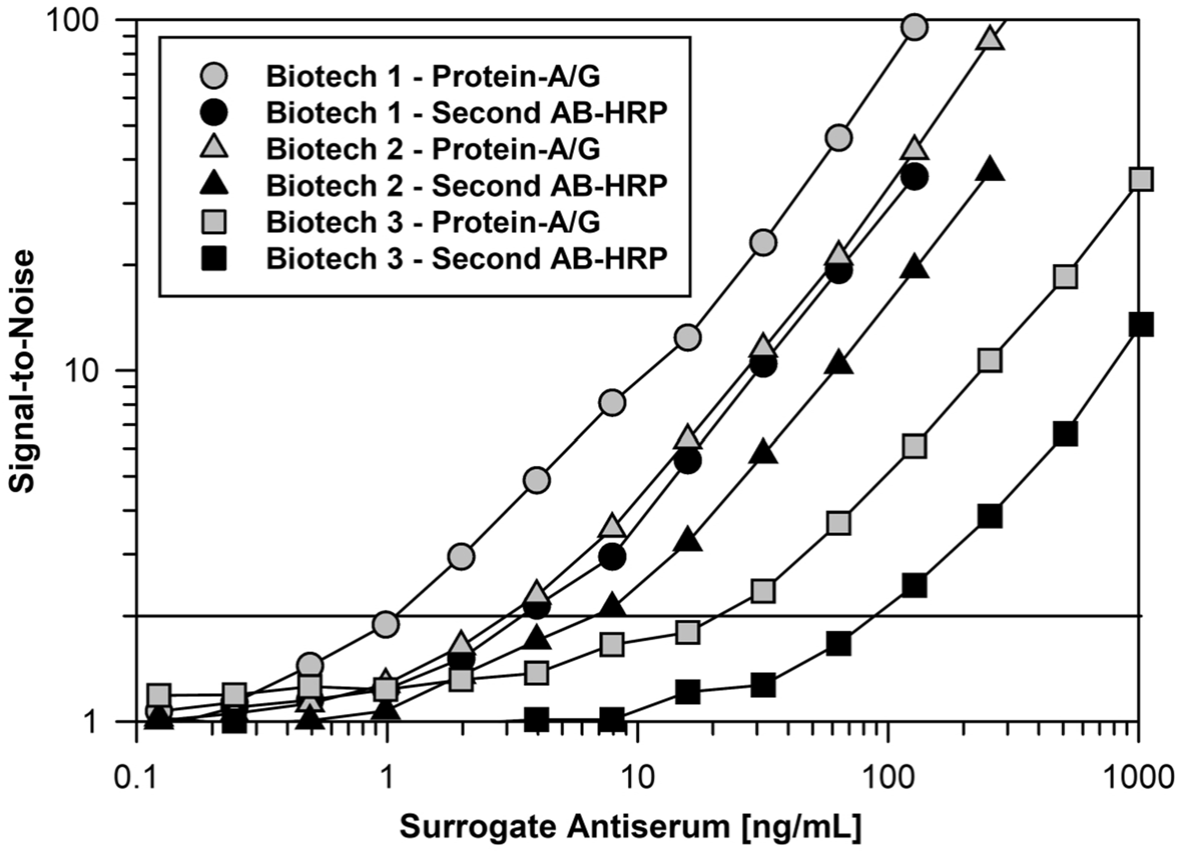
Comparison of serum immunoglobulin detection by optimized assay using SULFO-TAG labeled Protein-A/G versus conventional ELISA with horseradish peroxidase. MSD plates were coated overnight at 4 °C with 2 µg/mL of 3 different biotherapeutics. On the following day, the plates were washed and blocked with ChonBlock. Standard curves of surrogate MAbs specific to the various biotherapeutics were prepared in human serum and then diluted 1/100 in ChonBlock. The remainder of the assay was performed as described in the “Materials and methods” section using detection with 0.1 µg/mL of SULFO-TAG labeled Protein-A/G or a 10,000-fold dilution of HRP labeled Protein-A/G diluted in LowCross Buffer. For all three surrogate antibodies, the response ratios were appreciably greater when SULFO-TAG labeled Protein-A/G was used as the detection reagent with 2-times background responses occurring at about 1, 3, and 20 ng/mL, respectively.

Lastly to verify the new binding assay design with SULFO-TAG labeled Protein-A/G offers improved detection for serum ADA, we compared its performance to a conventional ELISA using detection with horseradish peroxidase (HRP) labeled Protein-A/G across the three different candidate biotherapeutic drugs. As shown in Fig. 5, we achieved ADA sensitivity at about a fourfold lower surrogate antibody concentration with SULFO-TAG labeled Protein-A/G relative to conventional ELISA with HRP detection.

**Figure 6:**
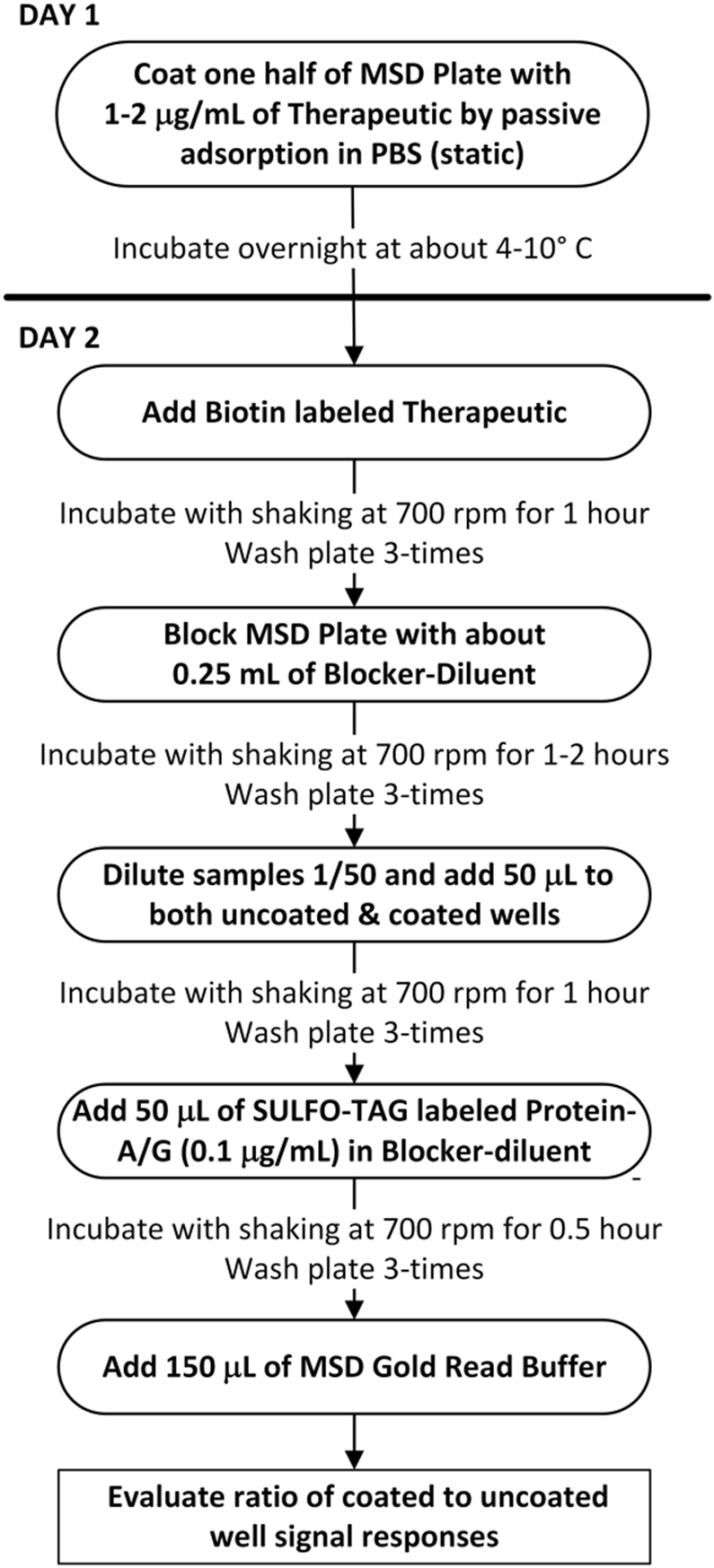
Workflow for Tier 1 screening ADA assay using SULFO-TAG labeled Protein-A/G.

A flow diagram for a screening assay utilizing uncoated and therapeutic-coated wells is described below in Fig. 6. As noted in the “Materials and methods” section, confirmatory assays are conducted in the usual way in which percent inhibition is calculated by comparing signal responses in the absence (buffer only) and presence of the added therapeutic-of-interest.

## Discussion

**Figure 7:**
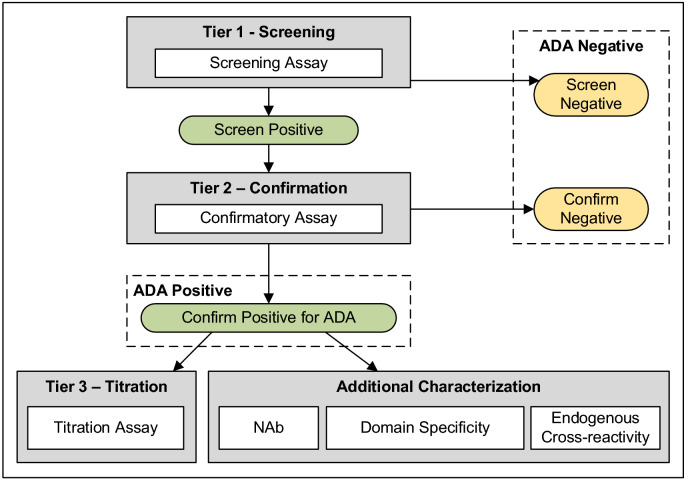
Generic workflow of the harmonized multi-tiered ADA sample testing scheme used to detect and characterize serum samples for the presence of reactive antibodies.

Today the detection and characterization of anti-drug antibodies consists of a harmonized multi-tiered testing approach that is outlined in detailed in key white papers^5–9^ and regulatory guidance documents^10–12^. In the first testing tier samples are evaluated in a screening assay. To minimize the risk of a false negative outcome for maximizing patient safety, responses from samples are compared to a statistically determined screening cut point that is computed to have a 5% false positive error rate (90% one-sided lower confidence limit)^10^. Samples with signal responses below the screening cut point are reported to be ADA negative and do not undergo further testing. Samples that generate a signal response ≥ the screening cut point are classified as being “potentially positive” for the presence of ADA and are submitted for testing in a tier 2 confirmatory assay. In the tier 2 assay samples are typically analyzed in the absence and presence of excess therapeutic to evaluate the extent of competitive inhibition. Samples that demonstrate a % inhibition < the confirmatory cut point that is set with a 1% false positive error rate (80% one-sided lower confidence limit)^10^ are termed False Positives and reported as being negative for ADA. Tier 2 negative samples do not undergo further testing. On-the-other-hand, samples that demonstrate a % inhibition ≥ the tier 2 cut point are categorized as being Truly Positive for reactive ADA and are submitted for tier 3 quasi-quantitative titer assessment. On a case-by-case basis additional ADA characterization may be dictated by the therapeutic’s structural attributes and its immunogenicity clinical risk assessment plan. A diagram of a standard scheme used for supporting clinical ADA testing is shown in Fig. 7.

Because of the growing diversity and structural complexity in modern biotherapeutics there is a need for continued evolution in ADA assay designs to support clinical assessments of immunogenicity. Of note, design options are needed to enable detection of serum ADA for candidate biotherapeutics that are not well-suited for testing by the widely used bridging assay design. Some examples include small peptide therapeutics, toxin-based therapeutics^34,35^, and fusion proteins, particularly those that are conjugated to a repeating polymer, such as polyethylene glycol^36,37^. The monomeric structural units within polyethylene glycol are known to favor formation of intra-molecular bridging and makes it as a poor candidate for ADA detection by a bridging assay^38^. To address this bioanalytical need, we initiated systematic refinement of the direct binding assay to adapt it for routine use in clinical testing to detect serum ADA to biotherapeutics lacking an immunoglobulin Fc domain. As such our aim for this manuscript is to report findings that establish the suitability of the direct binding assay using SULFO-TAG labeled Protein-A/G as one more design option that researchers can employ as part of their overall analytical armamentarium for ADA detection. We intend to report detailed results from pre-study method validation and in-study sample analysis for specific biotherapeutics by the direct binding assay design outlined herein in future manuscripts.

Even though the direct binding assay design has appeal for use in supporting clinical ADA testing because of its simplicity and potential for high throughput, it is characterized by some attributes that complicate its routine use for regulatory-compliant detection of ADA^5,8,9^. Chief among these are high matrix background signal responses that limit sensitivity, being prone to having false positives and, most notably, the requirement for using multiple species-specific immunoglobulin detection reagents, one for human and one for the animal surrogate positive control. Accordingly, we systematically optimized the direct binding assay with the aim of establishing a versatile design that would be suitable to accommodate today’s biotherapeutic diversity. First, we implemented SULFO-TAG labeled recombinant Protein-A/G as a sensitive detection reagent having broad reactivity to both human and other mammalian antibodies. Secondly, we identified several optimal blocker-diluents that maximize assay sensitivity by limiting the degree of non-specific binding. Lastly, we introduced a data transformation strategy involving biotherapeutic-coated and uncoated MSD wells that offers robust outlier identification, reduced skewness, and better normality that aids application of model-based parametric cut points for assigning plate specific screening cut points.

One practical operational issue that limits the appeal of the direct binding assay design is the requirement for using multiple species-specific secondary detection reagents^5,7–9^. This is because clinical ADA testing typically employs surrogate antibodies from animal species, such as rabbit or mouse, to support method development and validation, as well as in test sample analysis for run acceptance. To overcome this limitation, we adapted a SULFO-TAG labeled version of recombinant Protein-A/G as a sensitive reagent for universal detection of human immunoglobulin isotypes and surrogate positive control antibodies from commonly used animal species. Even though recombinant Protein-A/G has demonstrably higher reactivity towards IgG^31^, it reacts satisfactorily with other isotypes, including IgM and IgA, to allow its use as a broad-specificity detection reagent to support clinical ADA testing (Fig. 1). Furthermore, the specificity of labeled Protein-A/G is beneficial, as it will enable efficient transitioning of ADA assays from nonclinical toxicology to Phase I testing with minimal need for assay re-optimization. SULFO-TAG labeled reagents are used widely today in bridging assays for detection of ADA^8,9,22,23^. In addition, numerous publications have reported using Protein-A/G combined with other detection labels, such as horseradish peroxidase, for antibody detection in serology assays^39,40^. However, we believe our study is the first one to report the use of a SULFO-TAG labeled conjugate of recombinant Protein-A/G as the secondary detection reagent in a direct binding assay that is intended for supporting clinical ADA testing. In addition to offering excellent sensitivity to meet regulatory requirement for ADA detection, use of SULFO-TAG labeled Protein-A/G is convenient from a clinical lab testing workflow perspective because it eliminates the need for multiple species-specific detection reagents and uses the same electrochemiluminescence reader instrumentation that is used widely today in bridging ADA assays. Furthermore, in our experience when formulated with 0.2% BSA (w/v) as a carrier protein, SULFO-TAG labeled Protein-A/G demonstrates excellent long-term frozen stability at − 80 °C which is appealing for consistency in longitudinal testing of ADA. Despite its utility as a secondary detection reagent, SULFO-TAG labeled Protein-A/G suffers from one obvious cross-reactivity limitation. It is not suitable as a detection reagent if the biotherapeutic-of-interest possesses a Fc structural domain.

Heretofore, one factor that has complicated the application of SULFO-TAG labeled Protein-A/G as an ADA secondary detection reagent is the high-level of non-specific binding resulting from interference caused by the high concentration of unrelated serum immunoglobulins^29,41^. To solve this problem, we evaluated numerous different blocker-diluents to identify ones that yielded optimal assay signal-to-noise (i.e., ratio in the signal responses for biotherapeutic-coated wells / uncoated wells). In comparison to other blocker-diluents, we found ChonBlock to be particularly effective at limiting serum matrix non-specificity resulting from the avid binding of immunoglobulins and soluble immunoglobulin complexes to plastic surfaces^29,29,41–44^. When combined with an optimal blocker-diluent, such as ChonBlock, Assay Diluent or Blocker casein (Fig. 2), SULFO-TAG labeled Protein-A/G provides assay sensitivity that is suitable for routine detection of unwanted ADA at the clinically meaningful level of 100 ng/mL (Fig. 3)^10^. While the blocker-diluents recommended herein are typically the optimal ones, we have found that they can vary case-by-case depending on the structural attributes of the target therapeutic. For this reason, we recommend that a simple experiment be performed during method development to select the optimal blocker-diluent to use for a given therapeutic.

Today’s widely used multi-tiered ADA testing paradigm typically involves the use of a well-characterized negative control (NC) (i.e., serum pool from presumptive ADA negative samples) as the standard data transformation strategy in tier 1 screening assays^10,32,33^. This approach normalizes the signal responses from individuals relative to a common NC serum pool to reduce variability and aids computation of a plate specific cut point for reliable classification of test samples with a 5% false positive error rate^10–12^. However during this investigation, we noted an appreciable lack of normality and skewness from false positives (i.e., samples with extreme signal responses in the right tail relative to overall distribution, but lacking reactive antibodies). Upon further evaluation we determined that differences in inter-subject non-specific binding (i.e., binding response in uncoated MSD wells) was the principal cause for the observed outliers. Notably, previous studies have reported that inter-subject differences in non-specific binding of immunoglobulins and immune complexes to ELISA plastic surfaces can result in inaccuracy in reported results^41–45^. We resolved the increased inter-subject variability by implementing a modified data transformation approach for normalizing RLU signal responses. Similar to the one that is used in serology testing of infectious agents^41–44^, including SARS-CoV-2^46,47^, we implemented use of uncoated wells for each subject to control for the extent of non-specific binding. Accordingly, we adopted a normalization strategy in which the ratio of each test sample’s signal responses is determined after incubation in biotherapeutic-coated and uncoated MSD wells. The assay’s screening cut point is computed subsequently using the Log (coated wells/uncoated wells) data transformation. Unlike the common ADA testing practice used today in which results for test samples are evaluated by a plate specific cut point (i.e., cut point factor x mean negative control response), the modified approach described herein results in consistent assay cut point that is applied at the overall run level. We believe this alternative approach offers more reliable identification of biological outliers and, as reported in Table 1 and shown in Fig. 4, yields improved distributional normality and decreased skewness. Both attributes are desirable statistically for application of a parametric screening cut point. In contrast, a nonparametric cut point needs a much larger sample size to obtain reliable estimates at the 95th and 99th percentiles^33^.

In conclusion, the cumulative analytical innovations reported herein offers a versatile direct binding assay design strategy that is sensitive, operationally easy to execute, obviates the need for multiple species-specific detection reagents and is amenable to a testing a wide array of biotherapeutics that lack an immunoglobulin Fc domain. We believe this optimized direct binding assay design will find wide application in regulatory-compliant assays for detecting serum ADA to support clinical assessments of unwanted immunogenicity for contemporary biotherapeutics.

## Materials and methods

### Materials

Pierce recombinant Protein-A/G (#21186), Protein-A/G HRP conjugate (#32490) and Human IgA (31148) were purchased from ThermoFisher Scientific. Ninety-six-well Multi-Array Standard Bind MSD plates, MSD Gold SULFO-TAG NHS-Ester, and MSD Gold Read Buffer A were obtained from Meso Scale Diagnostics, LLC (Rockville, MD). Blocker casein (37528 or 37532) was purchased from ThermoFisher. ChonBlock ELISA blocker & sample diluent (#9068) was purchased from Chrondrex, Inc. (Woodinville, WA). Protein-free Assay diluent was purchased from Surmodics, Inc. (Eden Prairie, MN) and Candor LowCross buffer was obtained from Boca Scientific (Dedham, MA). ChromPure Human IgG (009-000-003) and IgM (009-000-012) were purchased from Jackson ImmunoResearch (West Grove, PA). The purified human IgG was purified by Size Exclusion chromatography using Sephacryl S-300 to remove a small amount of aggregated material. All other chemicals were high-quality, reagent- grade from either VWR or ThermoFisher. Sera from healthy adults was purchased from BioIVT (Westbury, NY). For collection of donor biological matrices, BioIVT obtains informed consent as required by the US Department of Health and Human Services regulations for the protection of human subjects (45 CFR §46.116 and §46.117) and Good Clinical Practice (GLP), (ICH E6).

Two different wash buffers were adopted for use in assays to support clinical ADA testing. Accordingly, they were designated as either Low Salt (VWR TBST, 25 mM Tris, 3 mM KCl, 140 mM NaCl, and 1.0% Tween 20, pH 7,4.) or High Salt (40 mM NaPO_4_, 500 mM NaCl, pH 7.4). The high salt wash buffer was prepared as a custom reagent by Boston BioProducts (Ashland, MA). Decisions about which wash buffer version to use were based upon the degree of stringency needed for efficient blank reduction. While low salt wash buffer is convenient operationally, high salt buffer yields higher signal-to-noise performance for some coated antigens.

### Preparation of SULFO-TAG Labeled Protein-A/G

After reconstitution in bicarbonate buffer (50 mM, pH 8.0), a solution of Recombinant Protein A/G (Thermo Scientific, cat # 77677) was prepared at a concentration at 2 mg/mL. MSD-Gold SULFO-TAG NHS-Ester (MSD, cat # R91AO-2) was suspended in dry DMF/water mixture, added promptly to the protein solution at a molar input ratio of about 12:1 (SULFO-TAG to Protein A/G), and allowed to react at ambient temperature in a sealed polypropylene vessel with gentle inversion to allow mixing. After about 2 h, the reaction was quenched by adding a small volume of amine-containing buffer, followed by gentle mixing for an additional 20 min. The SULFO-TAG labelled Protein A/G was purified by size exclusion chromatography using a HiPrep™ 26/40 column packed with Sephadex G-50 (fine) (GE, cat # 17-0042-01) and a mobile phase consisting of PBS supplemented with 5% sucrose and 0.05% Tween-20, pH 7.4. Detailed characterization of the purified conjugated product included concentration measurement by BCA Protein Assay, purity assessment by analytical HPLC-SEC and SULFO-TAG label molar incorporation ratio determination by Abs measurement at 455 nm. The conjugated protein was diluted into PBS containing 0.2% BSA buffer and stored in 0.1 mL aliquots in tightly sealed polypropylene vials at − 80 °C.

### Direct binding assay procedure for ADA detection

On the day prior to sample analysis, 96-well MSD plates were coated by passive adsorption with the biotherapeutic-of-interest at a concentration of about 2 µg/mL in PBS, sealed with Whatman Uniseal plate covers and incubated overnight in a common lab refrigerator at around 4 °C. Alternatively, biotinylated versions of the biotherapeutic were coated on a streptavidin-coated MSD plate for about 30–60 min at ambient temperature prior to use. On the next day, the plates were washed using a BioTek 405 automated plate washer for three cycles with 300 µL/well of wash buffer with the liquid aspirated completely after the last cycle.

The MSD plates were then blocked by adding 200–250 µL of ChonBlock. At this point all subsequent incubations were conducted at ambient temperature with plate shaking at approximately 700 rpm. After incubation for 1–3 h the MSD plates were washed again with another 3 cycles of wash buffer (300 µL/well). Prior to pipetting on MSD plates, test samples and quality control (QC) specimens underwent a MRD (minimal required dilution) ranging from 1:50 to 1:100. For example, specimens can be initially diluted 1:25 in ChonBlock or blocker casein, followed by a 1:2 dilution (equal vol.) into either ChonBlock (screening assays) or ChonBlock containing a 2X concentration of the biotherapeutic-of-interest (confirmatory testing). For confirmatory assays, samples were pre-incubated in a polypropylene 96-well plate to achieve competitive inhibition for approximately 1 h at ambient temperature. Aliquots (50 µL) of the diluted test samples and controls were added to duplicate MSD plate wells, sealed with Whatman Uniseal™ plate covers, and incubated for about 1 h. Following another round of plate washing, as described above, 50 µL of the SULFO-TAG labeled protein A/G detection reagent (diluted to 0.1 µg/mL in Low Cross Buffer (or Blocker casein) was added to each well followed by incubation for approximately 30 min. After washing and aspiration, 150 µL of MSD GOLD Read Buffer was added to each MSD well and the electrochemiluminescence (ECL) signal responses were quantified in a Meso Scale Quickplex 120 MSD reader.

### Data analysis and cut point statistics

Data analysis to compare ADA data transformation approaches was performed using JMP statistical software (SAS institute, ver. 15.2.1). Immunogenicity screening cut point factors were determined as described previously^33^. Initial estimates were obtained using Cut Point^+^, an on-line statistical tool for determination of ADA cut points (https://B2SLifeSciences.com/cut-point/).

## Abbreviations

ADA: Anti-drug antibodies
ECL: Electrochemiluminescence
HRP: Horseradish peroxidase
mAb: Monoclonal antibody
MRD: Minimal required dilution
MSD: Meso scale discovery
NC: Negative control
RLU: Relative luminescence units

## References

[1] Wang YM (2016). Evaluating and reporting the immunogenicity impact for biological products: a clinical pharmacology perspective. AAPS J..

[2] Shankar G (2015). The quintessence of immunogenicity reporting for biotherapeutics. Nat. Biotechnol..

[3] Chirmule N, Jawa V, Meibohm B (2012). Immunogenicity to therapeutic protein: impact on PK/PD and efficacy. AAPS J..

[4] Dingman R, Balu-Iyer SV (2019). Immunogenicity of Protein Pharmaceuticals. J. Pharm. Sci..

[5] Pineda C, Hernandez GC, Jacobs IA, Alverez DF, Carini C (2016). Assessing the immunogenicity of biopharmaceuticals. BioDrugs.

[6] US Pharmacopeia (USP). Immunogenicity assays: design and validation of immunoassays to detect anti-drug antibodies. *United States Pharmacopeia and the National Formulary* (USP-NF). USP <1106> 909 (2012).

[7] Mire-Sluis AR (2004). Recommendations for the design and optimization of immunoassays used in the detection of host antibodies against biotechnology products. J. Immunol. Methods..

[8] Wadhwa M, Thorpe R (2019). Harmonization and standardization of immunogenicity assessment of biotherapeutic products. Bioanalysis.

[9] Wadhwa M, Knezevic I, Kang HN, Thorpe R (2015). Immunogenicity assessment of biotherapeutic products: An overview of assays and their utility. Biologicals.

[10] US FDA Guidance for Industry. Immunogenicity testing of therapeutic protein products: Developing and validating assay for anti-drug antibody detection. https://www.fda.gov/media/119788/download/ (2019).

[11] Ishii-Watabe A (2018). Immunogenicity of therapeutic protein products: Current considerations for anti-drug antibody assay in Japan. Bioanalysis.

[12] European Medicines Agency. Guideline on immunogenicity assessment of biotechnology-derived therapeutic proteins. https://www.ema.europa.eu/en/documents/scientific-guideline/guideline-immunogenicity-assessment-therapeutic-proteins-revision-1_en.pdf. Accessed 1 Dec 2017.

[13] Hafeez U, Gan HK, Scott AM (2018). Monoclonal antibodies as immunomodulatory therapy against cancer and autoimmune diseases. Curr. Opin. Pharmacol..

[14] Sing S (2018). MAbs: A review. Curr. Clin. Pharmacol..

[15] Lu RM (2020). Development of therapeutic antibodies for the treatment of diseases. J. Biomed. Sci..

[16] Farkona S, Diamandis EP, Blasutig IM (2016). Cancer immunotherapy: the beginning of the end of cancer?. BMC Med..

[17] Weiner GJ (2015). Building better monoclonal antibody-based therapeutics. Nat. Rev. Cancer..

[18] Oldham RK, Dillman RO (2008). MAbs in cancer therapy: 25 years of progress. J. Clin. Oncol..

[19] Myler H (2019). Report on the AAPS Immunogenicity Guidance Forum. AAPS J..

[20] Partridge MA, Purushothama S, Elango C, Lu Y (2016). Emerging technologies and generic assays for the detection of anti-drug antibodies. J. Immunol. Res..

[21] Mikulskis A, Yeung D, Subramanyam M, Amaravadi L (2011). Solution ELISA as a platform of choice for development of robust, drug tolerant immunogenicity assays in support of drug development. J. Immunol. Methods..

[22] Wang Y, Luong M, Guadiz C, Zhang M, Gorovits B (2019). Addressing soluble target interference in the development of a functional assay for the detection of neutralizing antibodies against a BCMA-CD3 bispecific antibody. J. Immunol. Methods..

[23] Collet-Brose J (2016). Evaluation of multiple immunoassay technology platforms to select the anti-drug antibody assay exhibiting the most appropriate drug and target tolerance. J. Immunol. Res..

[24] Bivi N (2020). Development and validation of a novel immunogenicity assay to detect anti-drug and anti-PEG antibodies simultaneously with high sensitivity. J. Immunol. Methods..

[25] Niu H (2017). A biotin-drug extraction and acid dissociation (BEAD) procedure to eliminate matrix and drug interference in a protein complex anti-drug antibody (ADA) isotype specific assay. J. Immunol. Methods..

[26] Bourdage JS (2007). An Affinity Capture Elution (ACE) assay for detection of anti-drug antibody to monoclonal antibody therapeutics in the presence of high levels of drug. J. Immunol. Methods..

[27] Zoghbi J (2015). A breakthrough novel method to resolve the drug and target interference problem in immunogenicity assays. J. Immunol. Methods..

[28] Waritani T, Chang J, McKinney B, Terato K (2017). An ELISA protocol to improve the accuracy and reliability of serological antibody assays. Methods X..

[29] Terato K, Do CT, Cutler D, Waritani T, Shionoya H (2014). Preventing intense false positive and negative reactions attributed to the principle of ELISA to re-investigate antibody studies in autoimmune diseases. J. Immunol. Methods..

[30] Choe W, Durgannavar TA, Chung SJ (2016). Fc-binding ligands of immunoglobulin G: An overview of high affinity proteins and peptides. Materials.

[31] Thermo Scientific, Pierce Ig Binding Proteins (Protein A, G, A/G and L), https://www.thermofisher.com/order/catalog/product/21186#/21186.

[32] Shankar G (2008). Recommendations for the validation of immunoassays used for detection of host antibodies against biotechnology products. J. Pharm. Biomed. Anal..

[33] Devanarayan V (2017). Recommendations for systematic statistical computation of immunogenicity cut points. AAPS J..

[34] Bellows S, Jankovic J (2019). Immunogenicity associated with botulinum toxin treatment. Toxins.

[35] Benecke R (2012). Clinical relevance of botulinum toxin immunogenicity. BioDrugs.

[36] Hong L, Wang Z, Wei X, Shi J, Li C (2020). Antibodies against polyethylene glycol in human blood: A literature review. J. Pharmacol. Toxicol. Methods..

[37] Lee CC (2020). Structural basis of polyethylene glycol recognition by antibody. J. Biomed. Sci..

[38] Ehlinger C (2019). A generic method for the detection of polyethylene glycol specific IgG and IgM antibodies in human serum. J. Immunol. Methods..

[39] Jaturapaktrarak C (2020). Protein A/G-based enzyme-linked immunosorbent assay for detection of anti-Pythium insidiosum antibodies in human and animal subjects. BMC Res. Notes..

[40] Chow NA (2017). Development of an enzyme immunoassay for detection of antibodies against Coccidioides in dogs and other mammalian species. PLoS ONE.

[41] Terato K, Do C, Chang J, Waritani T (2016). Preventing further misuse of the ELISA technique and misinterpretation of serological antibody assay data. Vaccine..

[42] Haberland A, Müller J, Wallukat G, Wenzel K (2018). Antigen-free control wells in an ELISA set-up for the determination of autoantibodies against G protein-coupled receptors-a requisite for correct data evaluation. Anal. Bioanal. Chem..

[43] Cafruny WA, Heruth DP, Jaqua MJ, Plagemann PG (1986). Immunoglobulins that bind to uncoated ELISA plate surfaces: Appearance in mice during infection with lactate-dehydrogenase-elevating virus and in human anti-nuclear antibody-positive sera. J. Med. Virol..

[44] Kenny GE, Dunsmoor CL (1983). Principles, problems, and strategies in the use of antigenic mixtures for the enzyme-linked immunosorbent assay. J. Clin. Microbiol..

[45] Loeffler DA, Klaver AC (2017). Polyvalent immunoglobulin binding is an obstacle to accurate measurement of specific antibodies with ELISA despite inclusion of blocking agents. Int. Immunopharmacol..

[46] Bowsher RR, Devanarayan V (2020). Are lessons learned in setting cut points for detection of anti-drug antibodies also useful in serology assays for robust detection of SARS-CoV-2 reactive antibodies?. AAPS J..

[47] Yuen RR (2020). Novel ELISA protocol links pre-existing SARS-CoV-2 reactive antibodies with endemic coronavirus immunity and age and reveals improved serologic identification of acute COVID-19 via multi-parameter detection. Medrxiv.

